# Assessment of Outpatients' Knowledge and Adherence on Warfarin: The Impact of a Simple Educational Pamphlet

**DOI:** 10.22037/ijpr.2020.14766.12641

**Published:** 2019

**Authors:** Bahram Fariborz Farsad, Farzaneh Dastan, Jamshid Salamzadeh, Zahra Moghadamnia, Raha Eskandari, Fanak Fahimi

**Affiliations:** a *Rajaei Cardiovascular, Medical and Research Center, Iran University of Medical Sciences, Tehran, Iran. *; b *Department of Clinical Pharmacy, School of Pharmacy, Shahid Beheshti University of Medical Sciences, Tehran, Iran.*; c *Chronic Respiratory Diseases Research Center, National Research Institute of Tuberculosis and Lung Diseases (NRITLD), Shahid Beheshti University of Medical Sciences, Tehran, Iran. *; d *Food Safety Research Center, Shahid Beheshti University of Medical Sciences, Tehran, Iran.*

**Keywords:** Warfarin, Anticoagulation knowledge assessment, Anticoagulants, Thromboembolic disorders, Adherence, Patient education, Pamphlet

## Abstract

Warfarin is a critical medication that is broadly used for the treatment and prevention of thromboembolic disorders. Due to warfarin’s narrow therapeutic index, it is crucial that patients follow an appropriate dosage regimen. Patient knowledge is one of the most important factors to safe and effective use of warfarin. Due to the obvious risks of anticoagulants administration, evaluating patients’ awareness seems to be crucial. The purpose of this article was to evaluate the effects of intervention by an informative pamphlet on knowledge and adherence of patients who consumed warfarin. Two-hundred and fifty patients receiving warfarin were assigned to the study. They were asked to fill in the questionnaire. Then patients were provided with an educational pamphlet. In the second interview, patients filled the questionnaire again. Obtained data were assessed and analyzed by Excel software and SPSS version 18.0. Out of 250 patients who entered the study, 150 patients attended for the second interview. Data analysis revealed that out of 13 explanatory factors, only patients’ literacy level and income were the predictors which inversely correlated with the patients’ adherence (r = -0.44; *p* = 0.00040). Our educational intervention had a positive impact on patients’ knowledge regarding anticoagulation (*p* < 0.0001). Our findings revealed that a written informative pamphlet could effectively increase patients’ anticoagulation knowledge. Since, poorly literate patients had a lesser level of knowledge before and after educational intervention, it is recommended to develop appropriate educational programs especially designed for this group of patients.

## Introduction

Patient’s low-adherence is one of the greatest challenges in health care system. Poor adherence especially in medications with low therapeutic index could increase medication risks and treatment costs. Patients receiving warfarin therapy have difficulties maintaining sufficient adherence, which directly affects anticoagulation control (1).

Warfarin is a vitamin K antagonist that is the leading oral anticoagulation medication and it is broadly used to treat or prevent thromboembolic disorders. Warfarin has a narrow therapeutic index that requires frequent monitoring and proper patient adherence to achieve treatment goals (2).

Patients’ knowledge on warfarin therapy is indirectly proportional to risk of adverse events, mainly major bleeding, so it is challenging to keep patients’ treatment within the therapeutic index (3). It is crucial to instruct patients on the risks and the benefits of anticoagulation and make sure that they have acquired comprehensive knowledge on warfarin consumption, drug interactions, and regular monitoring. Moreover, non-adherence to warfarin therapy is linked to a more variable anticoagulation control which can be resulted from patients’ lack of knowledge (4). 

Although various educational methods were used, warfarin control remains frequently suboptimal (5). Bleeding complications are frequently caused by poor accuracy of warfarin usage (6). Patients who have received warfarin education by either written or verbal means showed a better control in terms of anticoagulation goals (7). Effective special strategies to deliver complicated information to improve patients’ knowledge about their medications are rarely evaluated (5). So we need more studies to assess these strategies. Face to face and multidisciplinary educational interventions seem to be proportional to a better success (8), specially because the quality of warfarin therapy in Iranian patients is poorer compared to European patients (9).

To address these concerns, we conducted a study among patients to assess the effectiveness amount of education by informative pamphlets on their adherence to therapy and anticoagulation control and to further evaluate patients’ baseline knowledge on warfarin.

## Experimental

We conducted a survey among patients referring to the anticoagulation clinic at a tertiary referral hospital, Tehran, Iran. The hospital has a total capacity of 573 inpatient beds with approximately 220000 patients visiting the hospital every year. The study was approved by hospital ethics committee.

Data collection tool was a questionnaire which was designed and validated based on Anticoagulation Knowledge Assessment (AKA) questionnaire (10). The AKA test was applied to evaluate patient knowledge in 9 content areas. The questionnaire is a 29 item, multiple-choice instrument (11) and consistent with best practices for assessing validity and reliability of patient knowledge instruments (12). Instrument validity was evaluated by using a Rasch model analysis of data. The instrument was designed to be self-administered at a 7th-grade reading level. The AKA instrument has different levels of cognitive difficulty (13). Overall, the AKA instrument seems to adequately measure patient knowledge in a variety of content areas using questions of modest cognitive difficulty (11).

We translated the questionnaire into the Persian language that was in compliance with the comprehensive standard protocol described in translation guidelines (14, 15). Three medical professionals reviewed the questionnaire and confirmed the translation. Moreover, the questionnaire was reviewed and approved by an English/Persian instructor to guarantee the correctness of both questionnaires and understanding by non-medical people.

Following that, three expert clinical pharmacists and one cardiovascular specialist confirmed the questionnaire. The questionnaire comprised 36 questions. Fifteen items evaluated patients’ demographic data and medical history including age, sex, level of literacy, field of education, employment, insurance status, monthly income, number of family members, history of warfarin use, dosage of warfarin, concurrent illnesses and medications, ease of access to information and previous knowledge of warfarin therapy (either by self-education or educated by healthcare team). Five questions assessed patients’ adherence to warfarin therapy and 16 questions were designed to evaluate patients’ knowledge on warfarin.

Our inclusion criteria for participating in the study were: adults (aged > 18 years old) receiving warfarin therapy, filling out the questionnaire independently, physical and psychological well-being to accurately answer the questions, and living in Tehran with the aim of facilitating further follow-up. Following preliminary assessment during the first visit, participants were provided with a standard educational pamphlet including information such as: warfarin indication, dosage, adverse effects, and drug-drug or food-drug interactions. The pamphlet was written in a simple language to improve patients with low-education understanding of medical issues. The pamphlet had 11 important points answered about warfarin and at the end of the pamphlet, instructions were added for patients in special cases such as pregnancy and traveling. Afterwards, the AKA was re-administered during the next visit to the clinic. The mean ± SD of the time between the two knowledge evaluations was 14 ± 2 days.

Data analysis was performed using the SPSS software, version 18.0 (SPSS, IBM Inc., Chicago, IL, USA). Rank correlation test (Spearman’s and Kendall’s), Mann-Whitny, Kruskal-Wallis, Chi-square Test and Fisher’s exact test were used to analyze the data. Also, a ridge regression model was developed for multivariate analysis.

## Results

Out of 250 patients who entered the study during the first stage, 150 patients attended for the second interview, among them 99 (66%) were females and 51 (34%) were males with the mean ± SD age of 52.97 ± 13.76 years. Eleven explanatory factors were listed in Table 1. Preliminary results demonstrated a significant correlation between patients’ adherence and the level of education (*p* = 0.0003), employment (*p* = 0.007), insurance status (*p* = 0.03), monthly income (*p* = 0.0001), and previous knowledge on warfarin therapy (*p* = 0.02). Patients with higher level of education had lower adherence. On the other hand; unemployed, uninsured patients, and lower-income patients had higher adherence to warfarin therapy. Due to co-linearity between explanatory factors, to build the final model, a multivariate ridge-regression analysis containing all predictors, which were significantly associated with the warfarin adherence based on the preliminary analyses, was performed. The final model showed that there is only a negative correlation between level of education and income with the warfarin adherence (r = -0.44; *p* = 0.0004) (Table 2). Furthermore, patients with higher level of education had higher previous knowledge on warfarin therapy before intervention (*p* ≤ 0.0001; r = 0.49).

After the intervention and educating patients with prepared pamphlets, patients’ knowledge on warfarin was significantly improved; mean patients’ score on knowledge about warfarin before intervention and after the intervention was 9.32 ± 2.77 and 13.46 ± 2.55, respectively (*p* ≤ 0.0001).

Further analysis of data revealed a significant positive correlation between level of education and patient knowledge on warfarin therapy after intervention (*p* = 0.002; r = 0.41). 

**Table 1:** Correlation between patients’ knowledge and the variables

Independent Variables	*P*-value (before intervention)	*P*-value (after intervention)
**Age**	0.05	0.6
**Sex**	0.66	0.99
**Level of literacy**	<0.001	<0.001
**Employment**	0.24	0.06
**Insurance status**	0.2	0.002
**Income**	<0.001	0.004
**Concurrent illnesses**	0.65	0.18
**History of warfarin**	<0.001	0.14
**Ease of access to warfarin**	0.57	0.07
**Previous knowledge regarding warfarin therapy**	<0.001	-
**Receiving information from health care personnel**	0.03	-

**Table 2:** Correlation between patients’ adherence and independent variables

Variable	*P*-value
**Level of literacy**	<0.001
**Employment**	0.007
**Insurance status**	0.03
**Monthly income level of literacy**	<0.001
**Concurrent medication employment**	0.36
**Previous knowledge regarding warfarin therapy**	0.02
**Knowledge on warfarin therapy (according to patients' score based on questionnaire)**	0.06
**Concurrent illnesses**	0.1
**Ease of access to medication**	0.55

## Discussion

The results of our cross-sectional, single center study demonstrated the beneficial impact of educational intervention that could significantly improve knowledge of the patients on anticoagulant therapy (*p* = 0.0001). Health literacy is an important factor that individuals can achieve and understand the information and services necessary to make appropriate decisions involving their health. For a potentially hazardous medication such as warfarin, it is vital to optimize patients’ understanding and involvement in clinical decision-making (16). Caregivers could achieve better outcomes if their patients are more involved and knowledgeable. Thus, identifying factors responsible for non-adherence to therapies could potentially lead to patient care improvement. A study of 143 patients taking warfarin demonstrated that patients with lower numeracy skills had more difficulty in controlling anticoagulation. However, we restricted this study to patients who spoke Persian, therefore, we failed to adjust for potential confounders such as ethnicity, gender, and duration of anticoagulation (17). 

The results of this study demonstrated that between 50-80% of older patients had inadequate knowledge about basic details of warfarin therapy. Demographic factors such as lower family income and limited health literacy affected patients’ warfarin knowledge and were inversely associated with patients’ adherence to therapy. It can be elucidated from these results that patients who have lower level of education are more likely to follow healthcare provider orders, but patients who are more educated and earn more money are less likely to comply with rational use of medication; higher level of literacy was correlated with lower patient’s adherence (*p* = 0.0004; r = 0.44). The most likely explanation is that more literate people might be more reluctant to listen and follow new directions. These results are reflective of previous studies; in a study done by Jing Jin *et al.* subjects with lower level of education had better adherence (18). Furthermore, a similar study done in UK demonstrated that patients without formal education had a better adherence to cholesterol lowering medications (19). This may be similar to diabetes care, where disease management program appeared to have greater benefit in patients with lower level of literacy (20).

In addition, access to warfarin education is often suboptimal in different practice settings. Lack of provider time to teach as well as lack of multi-disciplinary approach to teaching in busy outpatient healthcare settings were as major barriers to patient education in the present study. Educational strategies and resources such as information provided by booklets, pamphlets, and videos for home use and group classes can be used to improve patients’ knowledge. Providing individual classes for patients including patient knowledge assessment of oral anticoagulation therapy enhances outcomes (5).

Patients with lower level of literacy were more likely to answer incorrectly to most questions addressing warfarin-related knowledge, demonstrating lower level of health literacy. This was consistent with Rolls Chanelle A *et al.* findings (21). Our results were similar to previous finding by Hu *et al.* who reported higher knowledge in drug therapy among patients with higher level of education (22). Anticoagulation clinics can modify anticoagulation treatment. Due to diversities in patients’ understanding, periodic follow-ups of the patients may reduce potential inappropriate warfarin control (23). 

There is no direct trial of clinician-patient communication and/or the quality of shared decision-making experienced by the patients. Our measure of adherence was by self-report, which may be subject to evoke bias, social desirability, and mismeasurement (24). Our technique to promote patient’s knowledge was simple and clear. According to our results, it was an appropriate method for this purpose; however, alternative methods of patient education need to be investigated, such as: video-assisted instruction, improvements in verbal communication, or visual instruction techniques.

As we anticipated, the current study highlights the need to assess patient’s understanding of the importance of the instructions that should be followed to achieve appropriate anticoagulation therapy as well as to ensure that patient is educated about the therapy’s risks and benefits. 

Due to the small sample size, we could not assess the thromboembolic or hemorrhagic complications associated with anticoagulation knowledge and/or health literacy. However, because similar studies demonstrated that anticoagulation control is strongly associated with hemorrhagic complications of warfarin, it seems that INR was a reasonable alternative to assess hemorrhagic risk (25, 26). Finally, since we conducted the study in a single medical center serving our results might not reflect a diverse group of population. 
